# Supplementary material for: Mutations in PCYT2 disrupt etherlipid biosynthesis and cause a complex hereditary spastic paraplegia
Source: Brain. 2019 Oct 22;142(11):3382–97. doi: 10.1093/brain/awz291 (PMC6821184; doi:10.1093/brain/awz291)
Supplement: awz291_Supplementary_Data [file awz291_supplementary_data.zip › awz291-Suppl_data/Supplementary_Data4.zip!/Supplementary information 4 - plasma lipidomics.zip!/Plasma lipidomics/Control vs Patient3b/Statistical plots and data/Volcano plot.pdf]

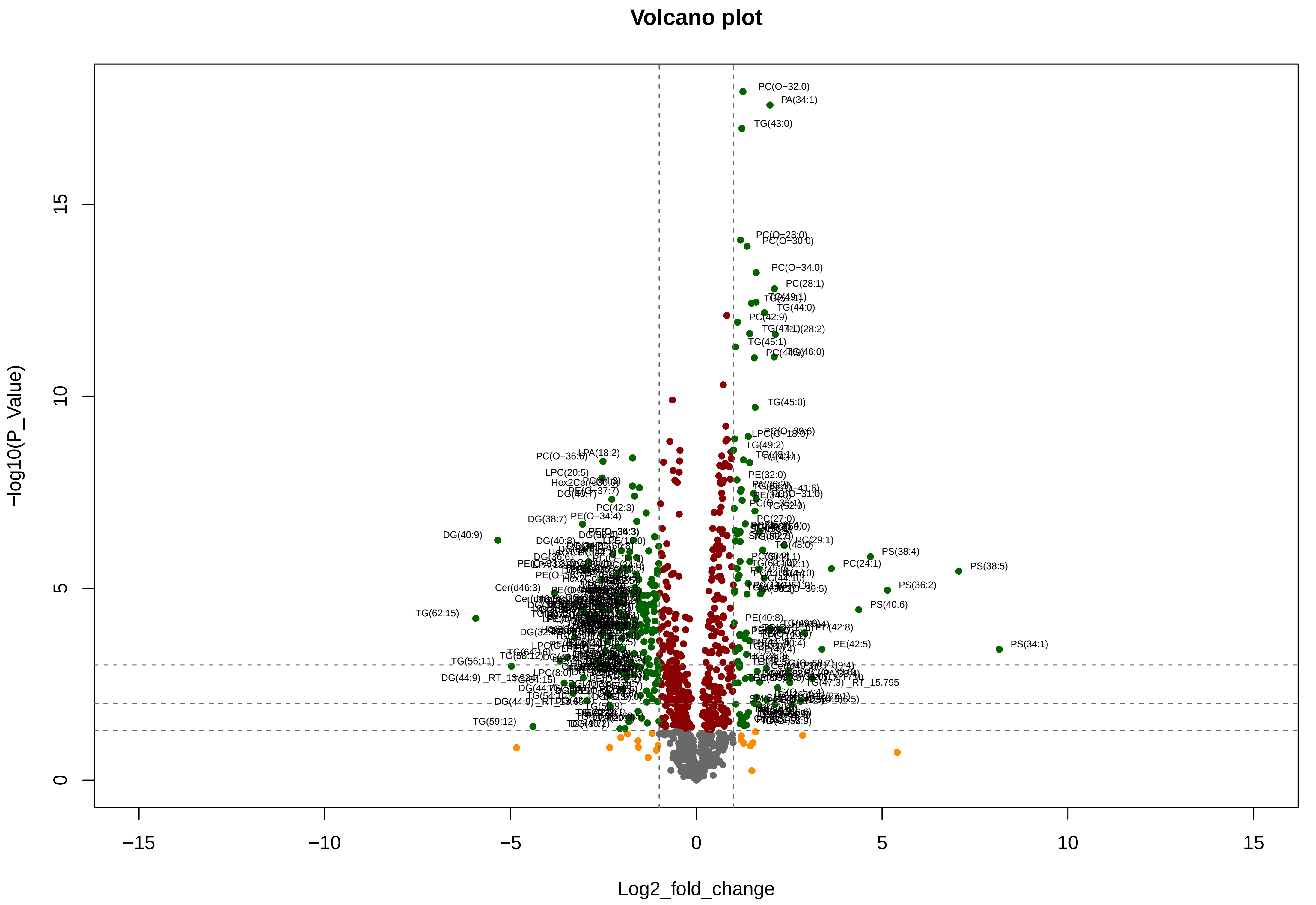
Volcano plot
−log10(P_Value)
Log2_fold_change
0
5
10
15
−15
−10
−5
0
5
10
15
PC(O−32:0)
PA(34:1)
TG(43:0)
PC(O−30:0)
PC(O−34:0)
PC(28:1)
TG(44:0)
PC(42:9)
TG(45:1)
TG(45:0)
TG(49:2)
PE(32:0)
PS(38:4)
PC(24:1)
PS(38:5)
PS(36:2)
PS(40:6)
PE(42:5)
PS(34:1)
PE(40:8)
TG(47:3)_RT_15.795
LPA(18:2)
PC(O−36:6)
LPC(20:5)
DG(40:7)
PC(42:3)
DG(38:7)
PE(O−34:4)
DG(40:9)
DG(40:8)
Cer(d46:3)
TG(62:15)
TG(56:11)
TG(58:12)
DG(44:9) _RT_13.924
TG(64:15)
DG(44:9)_RT_13.85
TG(59:12)
